# How Does Immunomodulatory Nanoceria Work? ROS and Immunometabolism

**DOI:** 10.3389/fimmu.2022.750175

**Published:** 2022-03-17

**Authors:** Lena M. Ernst, Victor Puntes

**Affiliations:** ^1^Vall d’Hebron Research Instiute (VHIR), Barcelona, Spain; ^2^Instiut Català de Nanociència I Nanotecnologia (ICN2), CSIC, The Barcelona Institute of Science and Technology (BIST), Universitat Autònoma de Barcelona (UAB), Barcelona, Spain; ^3^Institució Catalana de Recerca I Estudis Avançats (ICREA), Barcelona, Spain; ^4^Networking Research Centre for Bioengineering, Biomaterials, and Nanomedicine (CIBER-BBN), Instituto de Salud Carlos III, Madrid, Spain

**Keywords:** nanoparticles, nanoceria, inflammation, macrophages, immunemetabolism, metabolism, ROS - reactive oxygen species, entropy

## Abstract

Dysregulation of the immune system is associated with an overproduction of metabolic reactive oxygen species (ROS) and consequent oxidative stress. By buffering excess ROS, cerium oxide (CeO_2_) nanoparticles (NPs) (nanoceria) not only protect from oxidative stress consequence of inflammation but also modulate the immune response towards inflammation resolution. Immunomodulation is the modulation (regulatory adjustment) of the immune system. It has natural and human-induced forms, and it is part of immunotherapy, in which immune responses are induced, amplified, attenuated, or prevented according to therapeutic goals. For decades, it has been observed that immune cells transform from relative metabolic quiescence to a highly active metabolic state during activation(1). These changes in metabolism affect fate and function over a broad range of timescales and cell types, always correlated to metabolic changes closely associated with mitochondria number and morphology. The question is how to control the immunochemical potential, thereby regulating the immune response, by administering cellular power supply. In this regard, immune cells show different general catabolic modes relative to their activation status, linked to their specific functions (maintenance, scavenging, defense, resolution, and repair) that can be correlated to different ROS requirements and production. Properly formulated, nanoceria is highly soluble, safe, and potentially biodegradable, and it may overcome current antioxidant substances limitations and thus open a new era for human health management.

## Introduction

Inflammation and oxidative stress (OS), mediated by reactive oxidant species (ROS) overproduction, are strictly interconnected (1). ROS refers to various biogenic free radical molecules resulting from natural metabolism characterized by being highly oxidant. These free radicals are involved in different critical physiological processes, such as gene expression, signal transduction, growth regulation, and, significantly, inflammation, where high ROS concentrations are not only needed for the activation of inflammatory pathways but also to sustain the energetic demands of an inflammatory response (2). Therefore, from a theoretical point of view, antioxidant substances can both protect from oxidative stress (OS) and facilitate the resolution of pathogenic inflammation by inhibiting ROS-dependent inflammatory reactions and returning to homeostatic balance (3, 4).

The role of antioxidant substances became popular in the second half of the 20th century when Linus Pauling (1954 and 1962 Nobel laureate) developed the so-called orthomolecular medicine based on nutritional supplementation and high doses of ascorbic acid (5, 6). It resurfaced again in the 90s due to a large human study suggesting that vitamin E supplements could be associated with a reduced risk of heart diseases (7). During this period, other pre-clinical and epidemiological works also reported beneficial effects of antioxidant substances against chronic inflammation, neurodegeneration, and cancer (8). Subsequently, antioxidant therapies were evaluated in placebo-controlled trials involving tens of thousands of patients. Despite the pathophysiologic, epidemiologic, and mechanistic compelling evidence, these clinical trials have been, to date, mostly negative. This has been attributed to the non-drug-likeness of available antioxidant substances (9). These substances have high unspecific uncontrolled reactivity, poor solubility, and hence limited absorption profiles, low bioavailability, and low concentrations at the target site (10, 11). This has given rise to a pessimistic view of antioxidant therapies. Today, only a few antioxidant substances have reached clinical use. These include N-acetylcysteine for acetaminophen overdose, Edaravone for ischemic stroke, alpha-lipoic acid for diabetic neuropathy, some flavonoids (polyphenolic compounds present in dietary plants) for chronic venous insufficiency, as well as baicalein and catechins for osteoarthritis. Unfortunately, these treatments have not been fully satisfactory, and as a result, new approaches are being explored.

In these circumstances, new antioxidant mineral substances like nanoceria, displaying minimal toxicity to normal tissues while providing cellular protection from ROS-dependent oxidative damage, have attracted considerable attention as a potential therapeutic tool in preventing and treating oxidative stress-related diseases. With its mild but permanent ROS scavenging capacities and good pharmacology, nanoceria may overcome previous limitations and finally enable full antioxidant therapies in human health. Nanoceria has already demonstrated its ability to restrict inflammation in a large number of pathologies, based on their ability to reduce ROS levels and, consequently, most inflammatory mediators (12). Over the last decade, the beneficial effects of nanoceria treatment have been reported in various pre-clinical models, including cardiac diseases, diabetes, retinal diseases, gastrointestinal inflammation, liver inflammation, and cancer. In neurology, beneficial effects have been reported in pre-clinical models of Alzheimer’s disease, Parkinson’s disease, multiple sclerosis, traumatic brain injury, and brain ischemia, all conditions associated with high ROS production and neuroinflammation, reviewed in (13).

The underlying hypothesis is that by scavenging excess ROS, tissue is protected, metabolism is controlled, immune activation suffocated, and resolution of inflammation allowed. Thus, to understand how nanoceria works inside a biological system as an anti-inflammatory and immunomodulatory substance, it is essential to know how immune cells employ different metabolic pathways to sustain their energetic needs. For that, we focus on ROS production as a cause and consequence of inflammation and nanoceria ROS scavenging capacities. To propose a mechanism of action that results in effective and beneficial ROS scavenging and buffering, we need first to review the basics of metabolism and ROS production and immunometabolism, from a chemist’s point of view. Then we describe the nanoceria ROS reactivity towards unpaired electrons and free radicals. Finally, we consider pharmacological and production aspects for the proper development of medical formulations based on nanoceria.

## A Brief Overview on Metabolism, Mitochondria, and ROS

In our body, nutrients and oxygen are transformed into energy, water, carbon dioxide, and other by-products as ROS. The amounts of O_2_ consumed, and CO_2_ produced reflect the body’s metabolism and metabolized nutrients (14). In biological systems, energy is mainly provided by the controlled oxidation of carbohydrates and fatty acids, where the oxidizer is the limiting reagent. From a chemical point of view, oxidation of nutrients corresponds to the irreversible exothermic reaction of materials called fuels, consisting mainly of carbon and hydrogen, with an oxydizer. These oxidation reactions are more complex than they may seem. Initially, carbohydrates and fatty acids decompose to react with oxygen, forming unstable highly oxidant compounds called free radicals (ROS in our case). Then, these free radicals take C and H electrons, and most of the heat is released. Oxidation is completed when stable products are formed. It is interesting to note that incomplete oxidation leads to the release of highly reactive intermediates in the form of free radicals.

These nutrient oxidation chemical reactions are a fundamental part of metabolism. Metabolism can be divided into catabolism, the energy sourcing, extracting it from chemical bonds, breaking large molecules into smaller ones, and anabolism, the synthesis of complex molecules from simpler ones, using part of the produced energy. Regarding catabolism, three different basic catabolic pathways exist depending on the employed fuel (glucose or fatty acids derived from carbohydrates and lipids); oxidizer (O_2_/ROS), and combustion mode (aerobic or anaerobic). The produced energy is stored in the form of ATP (indeed, in the form of ATP/ADP gradients (15)) and heat. These pathways can be referred to as aerobic glycolysis, anaerobic glycolysis, and fatty acid oxidation.

Glycolysis is relatively efficient in aerobic conditions (high ATP production and low ROS production) and very inefficient in anaerobic conditions, where a lot of glucose is consumed, and a lot of ROS produced. In fatty acid oxidation (FAO), processing one palmitic molecule efficiently produces 129 ATP molecules, compared with 2 ATP molecules produced per molecule of glucose during anaerobic glycolysis, or 36 ATP molecules per molecule of glucose during aerobic glycolysis. FAO is accompanied by a slight non-pathological ROS overproduction when compared to aerobic glycolysis. Regarding power (*energy per unit time*), anaerobic glycolysis can produce ATP 100 times faster (16) than aerobic, thus providing the highest power to cells. FAO delivers energy at intermediate rates. These different catabolic pathways allow adjustments for the different cellular energetic requirements and needs, and as a consequence, biological responses can be controlled by targeting the energy supply. The following is important for our hypothesis: biological oxidation rate is adjusted by ROS concentration rather than oxygen concentration, which is more stable (constant) inside the body than ROS, and therefore the relevance of antioxidant substances.

**Figure 1:**
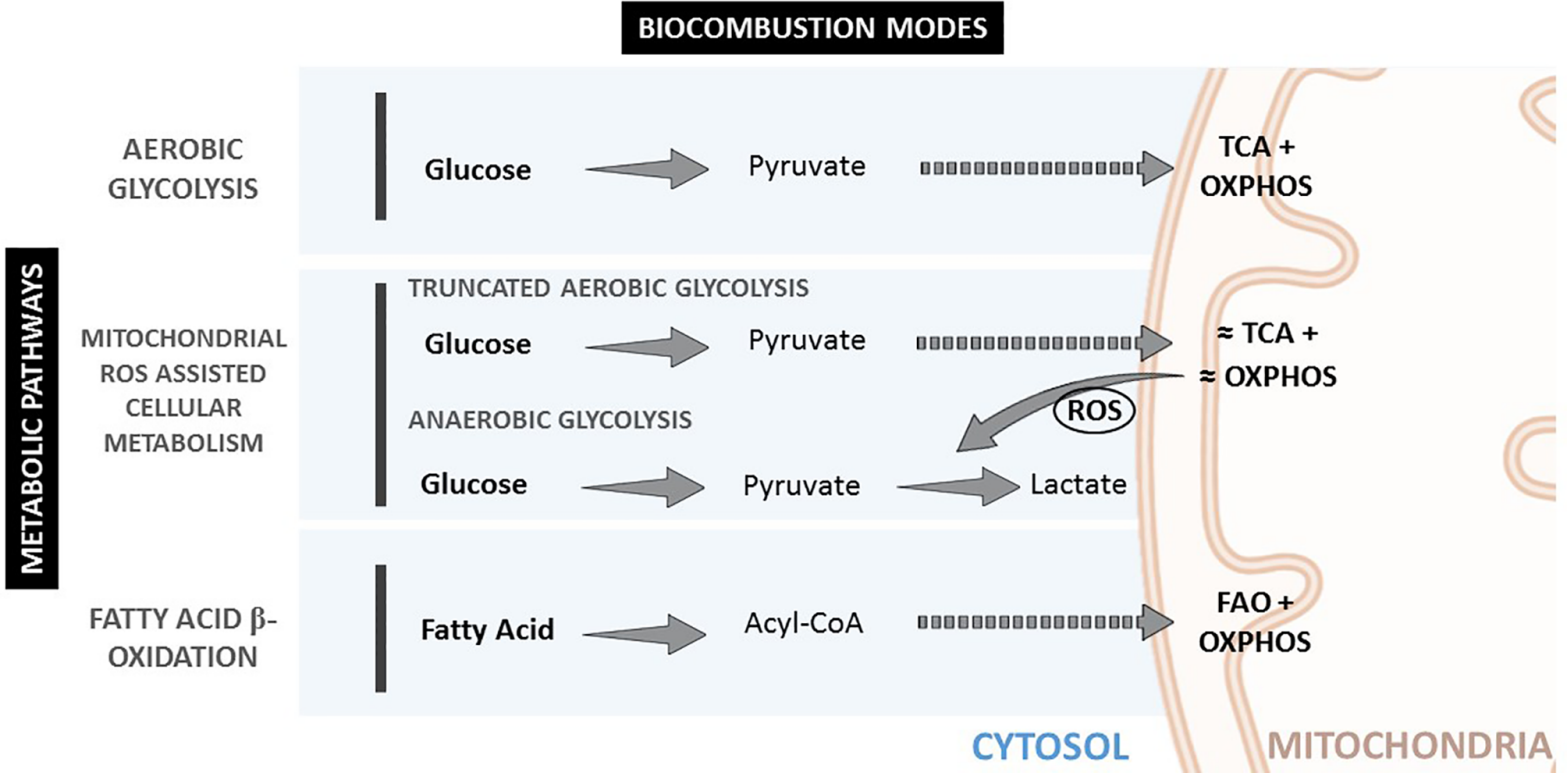
Metabolic pathways and biocombustion modes inside the cell.

These different cellular metabolic pathways can be observed by the lactate production, the expression of glucose transporters at the cell membrane, or mitochondria number and morphology (See **Figure 1** and **Table 1**). Mitochondria have been described as *cell power-houses*, converting nutrients in the form of glucose or fatty acids into energy in the form of ATP, and ROS. Mitochondria can rapidly adjust to the cell metabolic needs, and play a central role in bioenergetic and biosynthetic pathways. Increased energy demand is met by mitochondrial reproduction and fusion. In contrast, a decrease in energy demand results in the removal of superfluous mitochondria through fission and mitophagy.

**Table 1:** The three different mitochondria operating modes.

*CATABOLIC PATHWAY*	*Fuel*	*Oxidizer*	*Oxydation mode*	*Mitochondria shape*	*Mitochondria S-to-V ratio*	*Power suppy*	*ROS production*
***Aerobic Glycolysis***	Glucose	Oxygen	Aerobic	Elongate	Medium	Low	Basal
***Mitochondrial ROS assisted cellular metabolism***	Glucose	Oxygen &ROS	Anaerobic/ truncated aerobic glycolysis	Spherical	Low	High	High
***Fatty Acid β-oxidation***	Fatty Acid	Oxygen	Aerobic	Hyperfused	High	Medium	Medium

It has been described that ROS generate from the leakage of electrons in the mitochondria transport chain. Under normal conditions, the potentially harmful effects of ROS are successfully restrained by protective and reparative mechanisms. Natural antioxidant defenses may remove ROS either in a highly specific manner, e.g., by catalase, SOD, or glutathione peroxidases, or in a less specific way, with small molecules such as ascorbate, glutathione, alkaloids, or carotenoids (1).

From a chemical engineering perspective, mitochondria could be described as an internal combustion engine (17), transforming oxygen and organic fuels into energy and oxidized products such as CO_2_, H_2_O, and H_2_O_2_ (ROS). H_2_O_2_ exists in equilibrium with hydroxyl species (a model ROS), and it is a common source of free radicals. Therefore, for intervening in the operating mode of an internal combustion engine, one can either address the fuel supply (*e.g.*, ketogenic diets are known to both be anti-inflammatory and to restrict anaerobic glycolysis (18)), or the excess oxidizer (mainly ROS), *i.e.*, the target of antioxidant substances.

Changes in mitochondria membrane potential parallel mitochondrial morphology (oval, spherical, or elongated/branched), which determines oxygen supply rate and, consequently, activity (19). A high mitochondria surface-to-volume ratio (elongated/branched) allows the oxidation of high dense fuels as FA. A low surface-to-volume ratio (spherical) will result in the lack of oxygen and the production of highly reactive oxidation intermediates. Consequently, considering the mitochondria as an internal combustion engine where the oxygen is supplied through the membrane to the fuel inside it, the surface-to-volume ratio is directly proportional to the oxygen provision rate. Thus, higher mitochondrial membrane polarization implies a higher surface-to-volume ratio and higher oxygen provision to the fuel for oxidation. Mitochondria display an oval shape most of the time, providing a sufficient O_2_ supply for pyruvate oxidation in the TCA cycle and OXPHOS. In contrast, reduced O_2_ supply in spherical mitochondria, when the surface-to-volume ratio is minimal, induces incomplete oxidation (anaerobic glycolysis) and excessive ROS production, decreasing the cell redox potential and igniting the pyruvate in the cytoplasm. Later, mitochondria can fuse and expand their surface, increasing polarization and creating elongated and branched structures with a higher surface-to-volume ratio, allowing for FA consumption.

Accordingly, mitochondria reduce their number and surface area (depolarize) when the cell power requirement is high. In this case, glucose is fully oxidized and directly in the cytoplasm at high rates. This can be clearly observed in the connection between glucose transport and mitochondrial mode of work in conditions of OS (1), where stimulation of cellular glucose uptake is frequently concomitant to inflammation. Therefore, during anaerobic glycolysis, a high glycolytic flux and impaired oxidative phosphorylation are associated with increased ROS levels (20, 21). Interestingly, glucose uptake has also been described as an anti-ROS mechanism since excess mitochondrial ROS is consumed, burning the extra uptake of glucose (22). This is consistent with the fact that the oxidizer is the limiting reagent, and then the oxidizer concentration can be decreased by increasing the fuel supply. When ROS levels are too high and/or remain increased during a prolonged time, a vicious circle of ROS-stimulated glucose uptake and glucose-stimulated ROS production can be triggered. This pathological cycle can be broken by restoring mitochondrial ROS production to normal levels, a phenomenon that has stimulated interest in antioxidant therapies.

Antioxidant therapies should not eliminate all ROS. Under normal conditions, the potentially harmful effects of ROS are successfully restrained by protective and reparative mechanisms. Compartmented controlled ROS levels act as signaling molecules to mediate localized events *via* the oxidative modification of redox-sensitive mediators, which are needed at low doses for many normal biological functions, such as DNA replication and repair. However, they become toxic at high concentrations when the antioxidant cell defenses are overwhelmed. High ROS concentration induces OS, damaging phospholipids and DNA, inducing cell alterations, provoking mutations, and cell death. Consistently, abnormal ROS overproduction has been involved, directly or indirectly, in the pathogenesis and progression of many diseases, to the point that it is fair to ask if there is any disease without associated abnormal ROS production. And the answer seems to be no (23, 24).

ROS are highly reactive free radicals and, therefore, short-lived molecules, and can therefore be used by the cell to produce rapid and local responses. They are also challenging to target since they are highly *mutable* and quickly transform into different free radicals. Free radicals propagate in chain reactions. Thus, once a reactive free radical is generated, it can react with stable molecules forming new free radicals. For example, the unpaired electron transfer from O to N and S molecules produces reactive nitrogen species and reactive sulfur species, all highly oxidant. Chain termination occurs when two free radical species react with each other to form a stable, non-radical adduct. This, as discussed below, can be promoted by nanoceria. In addition, ROS, in the form of H_2_O_2_, easily cross biological membranes escaping their compartment and leaking into the tissue, diffusing in and outside cells.

All this is especially critical regarding the immune system (25), where the different immune responses have clearly different energetic needs and power requirements, especially during inflammation (26). For decades, it has been observed that immune cells transform from relative metabolic quiescence to a highly active metabolic state during inflammation (1). Inflammation requires high power consumption. This encourages the immune system to increase the production of cytokines and chemokines, phagocytosis, immune cell recruitment and activation. These changes in essential metabolic processes affect fate and function over a broad range of different timescales and cell types, making the expression of inflammation in different organs and conditions complex. In contrast, its basal metabolic pathways are very conserved. Therefore, it is possible to target metabolic processes by scavenging ROS during an immune response, modulating thus immune activity.

**Figure 2:**
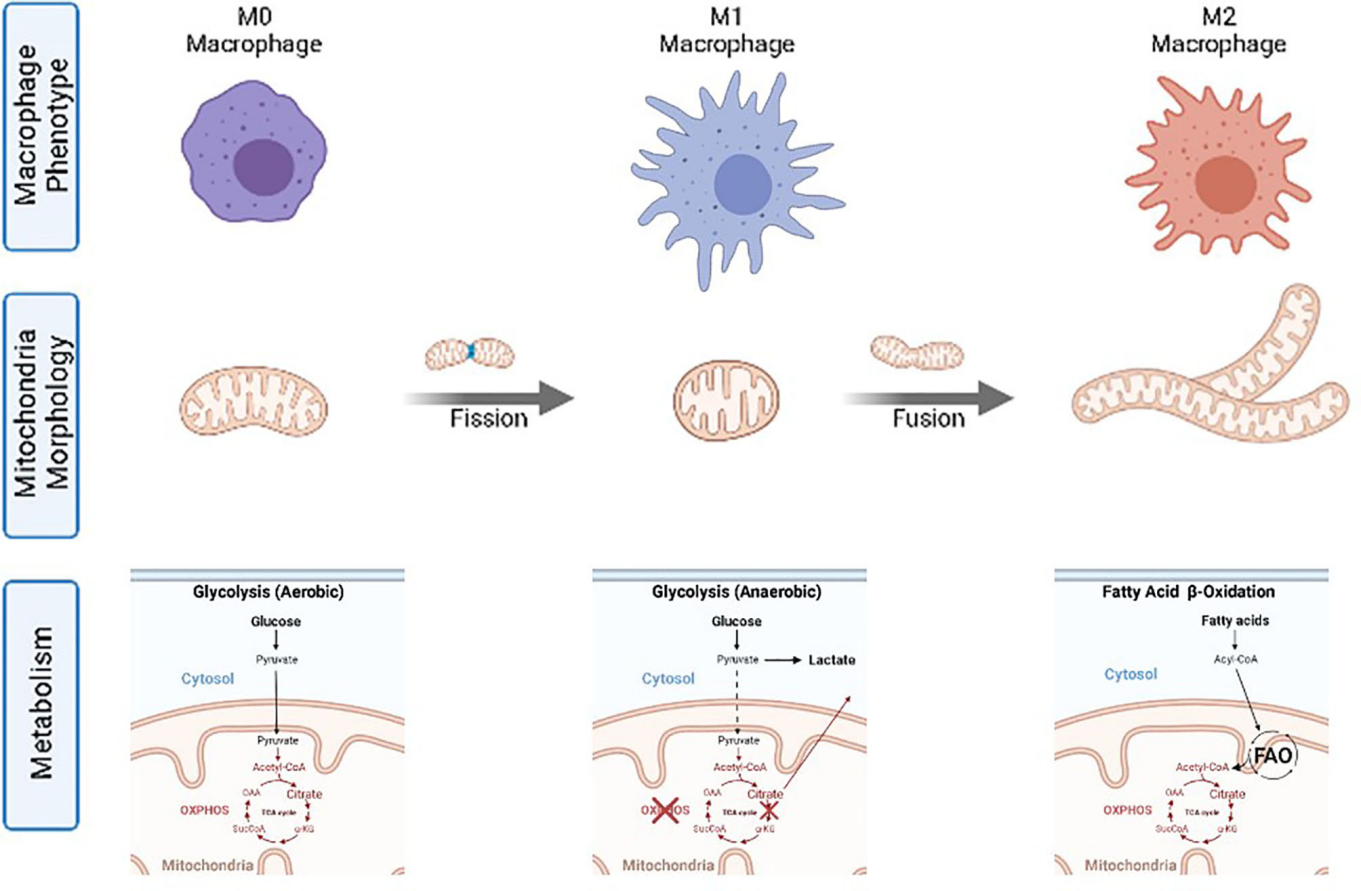
Macrophage phenotypes, mitochondria morphology and corresponding catabolic pathways.

Accordingly, macrophages, fundamental cells of the innate immune system responsible for detecting, categorizing and eliminating pathogens or aberrant cells, tissue repairing, development, and resolution of inflammation (27) follow the same metabolic trends. Macrophage functions can be grouped into three well-described macrophage functional phenotypes (also called cellular polarization) (28): M0 for resting, quiescent, macrophages; M1 for classical pro-inflammatory activation; and M2 for alternative activation when resolving an inflammatory reaction and promoting tissue repair (29). It has been independently and repeatedly observed that M0 works on aerobic glycolysis, M1 on anaerobic glycolysis, and M2 on FAO, providing different amounts of power and different ROS concentrations in each case (2) (**Figure 2**). Thus, changes in the basic macrophage metabolism occur following immune activation, shifting from reliance on aerobic glycolysis to increased anaerobic glycolysis, or FAO. The different macrophage polarization energy processing has been studied under the concept of immunometabolism (30).

The final question is how to modulate the immunochemical potential. As high ROS concentrations are needed to sustain anaerobic glycolysis, the cell response can be controlled by controlling ROS concentration. Modulating ROS should change the cell chemical potential, inducing metabolic adjustments and phenotypic changes towards inflammation resolution and homeostasis restoration in the case of macrophages. For that, ROS flooding the tissue and mutating have to be removed in many forms from everywhere, for a sufficiently long period of time, conditions that can only be achieved at unrealistically high doses with currently available substances and traditional molecular medicine.

## The Nanoceria Immunomodulatory Mechanism

Nanoceria has recently raised as an anti-inflammatory agent working very well in a wide variety of disease models. Indeed, cerium and other rare-earth compounds have been employed in medicine since the 19th century (31–33). The first reported use was that of Ce^3+^ oxalate as an antiemetic agent during pregnancy, reported by the Scottish doctor J.Y. Simpson in 1854. Subsequently, it came to be prescribed for other gastrointestinal disorders. Indeed, it gained unaccountably rapid and widespread popularity to treat sickness and coughing, and other nervous disorders such as chorea and epilepsy. Its fall into oblivion was almost as quick as it rose and for equally unclear reasons. It was said that: “Here, perhaps, is a good case of the right drug being used for the wrong reason” (34). Diversity of opinion regarding the therapeutic value of cerium oxalate has existed ever since. It is possible that the lack of a mechanistic description of its action, and the lack of standardized materials, were at the root of the controversy. Afterward, in the late 1950s, cerium oxide and phosphate tested in rats showed anti-inflammatory efficacy attributed to their dual valence state of oxidation (35). At these times, cerium and other lanthanide compounds found use as bio-imaging contrast agents in light and electron microscopy for the *in situ* detection of oxidase (36, 37) and phosphatase (38) activity since oxidized cerium precipitates in the form of highly electron-dense NPs. Later, in 1999, Telek et al. (39) used cerium chloride for the *in vivo* histological detection of oxygen-derived free radicals in inflammatory conditions, and observed anti-inflammatory effects. Although the precise mechanisms were not elucidated, the report hinted at a possible role of cerium precipitates in the observed decrease of ROS concentration. Besides, during the 20^th^ century, nanoceria was extensively developed in the petrochemical and automobile industry as catalysts. Currently, nanoceria is also employed as a polishing agent, as a glass constituent to prevent solar discoloration, and in coatings to protect metallic materials from corrosion. Its promising past having been forgotten, the nanoceria biomedical potential was re-discovered in Virginia Tech less than two decades ago, when it was observed that nanoceria of less than 20 nm prolonged the lifespan of brain cell cultures for periods of up to 6–8 months (40). This finding was described by Professor Berverly Rzigalinski, and collaborators Sudipta Seal, David Bailey, and Swanand Patil, as “somewhat serendipitous”, according to her words (41) since she was studying nanoceria as a drug carrier. Since then, many studies have been performed by them and others, and the potential therapeutical effects of nanoceria examined in many animal models of disease.

The first diseases subject to nanoceria treatment in pre-clinical models have been related to inflammation and diseases where antioxidants substances were previously assayed with positive results, from sepsis to age-related degeneration. Regarding sepsis, nanoceria has shown promising results regarding septic shock treatments where the mortality rate induced by LPS sepsis in rats decreased from 73% to 11% (34, 42, 43). Indeed, nanoceria has also been proposed to counteract the lethal effects of cytokine storms in COVID-19 patients (44). Regarding aging (45), nanoceria has shown significant protective effects in age-related diseases such as retinal degeneration (46), Alzheimer’s, and Parkinson’s (47, 48). Nanoceria has also demonstrated a positive impact in metabolic disorders, rather orphan of treatment, such as the metabolic syndrome or non-alcoholic fatty liver disease (NAFLD) (49), where ROS contribute to the initiation and progression of the disease. Another prominent example of metabolic disorder related to disease is cancer, illustrated by the Warburg effect and the metabolic reprogramming of cancer and other cells in tumor microenvironments, where anaerobic glycolysis is favored (44), suggesting that proliferation *contra naturam* costs extra energy. Another field where nanoceria could be beneficial is regenerative medicine. One of the biggest challenges in regenerative medicine and tissue engineering is to deal with inflammation. Typically, the tissue to reconstruct is under oxidative stress due to tissue damage that impedes proper regeneration. In this regard, in a partial hepatectomy animal model, rats treated with nanoceria showed a significant increase in liver regeneration compared with controls (50). Similarly, in an acetaminophen overdose experimental model, nanoceria and N-acetyl-cysteine treatments decreased early liver damage. However, only nanoceria was associated with a significant increment of hepatocellular proliferation (50).

The nanoceria chemical formula is commonly written as CeO_2_ since its primary oxidation state is Ce^4+^. Nevertheless, defects in the crystal structure are usually present at the nanoscale, and some Ce ions present a Ce^3+^ instead of a Ce^4+^ valance state. Having Ce^3+^ instead of Ce^4+^ induces a deficiency of positive charge, compensated with oxygen vacancies, usually occurring at the NP surface. The Ce^3+^ concentration in the NP, hence oxygen vacancies and redox activity, increases as the NP size decreases, achieving its maximum capacity and thermodynamic stability at diameters of a few (2 to 5) nm (50, 51). Notably, the nanoceria cubic fluorite crystal structure is preserved while Ce^4+^ is reduced to Ce^3+^ and oxygen vacancies formed. Consequently, Ce^3+^ can be easily re-oxidized (recycled) to Ce^4+^ and the vacancy covered, completing thus its catalytic loop.

The observed competitive nanoceria advantages, such as its electron sponge effect, catalytic behavior, and potential biodegradability, should be looked for in its electronic structure. Metallic cerium has an electronic configuration [Xe] 4f^1^5d^1^6s^2^, Ce^3+^ has an electronic configuration [Xe] 4f^1^5d^0^6s^0^, and Ce^4+^ has an electronic configuration [Xe] 4f^0^5d^0^6s^0^, indicating that the 4f electron is the labile one. The main difference of rare earths from other elements is to have these 4f orbitals, whose electrons are shielded by 4d and 5p orbitals. This orbital shield makes 4f electrons weakly bound to the nucleus, allowing for the Ce^3+^/Ce^4+^ tautomery. Thus, the two oxidation states of the cerium element in the face-centered cubic crystal lattice make possible the formation and occupation of oxygen vacancies essential to its oxygen (electron) buffering capabilities, and thus its ability to act as a catalyst for both oxidation and reduction reactions.

Nanoceria has been described as an antioxidant and anti-inflammatory agent since it produces effects similar to those substances. By measuring the nanoceria electronic structure in real-time by X-ray absorption spectroscopy during the catalytic degradation of H_2_O_2_, a rapid uptake of electrons by the NPs was evidenced, followed by a later and slower release of these electrons, and corresponding pH modifications, in such a way that nanoceria was described as electron sponges (52). A labile unpaired electron from the free radical can be passivated by either pairing it with another electron (antioxidants provide this electron), or removing it (by antireducers), with opposite effects on pH. Thus, nanoceria is antireducer (Ce^4+^ to Ce^3+^), and antioxidant (Ce^3+^ to Ce^4+^) during recycling, providing a high capacity to remove excess ROS from its surroundings. Normally, substances that uptake electrons are called electron sinks or antireducers, rather than antioxidants, even if the ROS scavenging effects are similar. Such molecules, antireducers, are ubiquitous in nature, especially in the photosynthesis reaction chain, where the generation of ROS by-products is higher than during metabolism.

Taking the hydroxyl molecule as model ROS, the oxygen atom in the OH· molecule is surrounded by 7 electrons in its valence band, and it is unsatisfied, longing for 8 (the octet). This situation can be overcome if the unpaired electron is removed, and Ce^4+^ passes to Ce^3+^, and the OH· becomes ½ O_2_ plus the formation of H^+^ (equation 1). Alternatively, the OH· molecule may get an electron from the nanoceria, and Ce^3+^ recycles to Ce^4+^, and the OH· transforms into OH^-^ (equation 2). Thus, the nanoceria catalytic cycle, with corresponding pH modifications and oxygen generation, can be described as follows:


(1) $$\text{C}{{\text{e}}^{4+}}_{\left(\text{s}\right)}+\text{OH}\cdot_{\left(\text{I}\right)}->\text{C}{{\text{e}}^{3+}}_{\left(\text{s}\right)}+1/20_{2(\text{g})}+{{\text{H}}^{+}}_{\left(\text{I}\right)}$$

(2) $$\text{C}{{\text{e}}^{3+}}_{\left(\text{s}\right)}+\text{OH}\cdot_{\left(\text{I}\right)}->\text{C}{{\text{e}}^{4+}}_{\left(\text{s}\right)}+\text{O}{\text{H}}^{-}{\;}_{\left(\text{I}\right)}$$

If we add the two equations:


(3) $$2\text{OH}\cdot_{\left(\text{I}\right)}->{\text{H}}_{2}{\text{O}}_{\left(\text{l}\right)}+1{/20}_{2(g)}$$

Accordingly, when mixing nanoceria and H_2_O_2_, oxygen generation is clearly observed in the form of vigorous bubbling. Note that oxygen generation by nanoceria could be related to their observed pro-angiogenic properties (53).

However, we have to take two other equations into account. At low OH· concentration, reaction (2) can be outcompeted by (4):


(4) $$\text{C}{{\text{e}}^{4+}}_{\left(\text{s}\right)}+1/{2O}_{2(\text{g})}+{{\text{H}}^{+}}_{\left(\text{I}\right)}->\text{C}{{\text{e}}^{4+}}_{\left(\text{s}\right)}+\text{OH}\cdot_{\left(\text{I}\right)}$$

and the ROS scavenging activity stopped, or even its production promoted. Additionally, at low ROS and O_2_ concentrations, the Ce^3+^ ion, which is soluble at pH below 8 (see Pourbaix diagram in SI), may dissolve away from the NP (indeed, it is the Ce^4+^ crystal structure that holds the Ce^3+^ soluble ions in the solid phase), and nanoceria slowly disintegrates, (equation 5).


(5) $$\text{C}{{\text{e}}^{3+}}_{\left(\text{s}\right)}->\text{C}{\text{e}}^{3+}{}_{\left(\text{I}\right)}$$

In other words, nanoceria can uptake a limited number of electrons maintaining their fluorite crystal structure and NP integrity. Due to the water solubility of Ce^3+^ ions and an increasing concentration of oxygen vacancies, if Ce^3+^ ions are not recycled to Ce^4+^, at some point, the fluorite crystal structure cannot be maintained, and the NP disintegrates.

The combination of these equations makes nanoceria act as a redox buffer. Free radicals have to be continuously supplied to the NP surface to allow their combination into more stable species. When the ROS concentration is low, and this condition cannot be fulfilled anymore, the NP loses its catalytic activity, and reactions 1 and 4 combine instead of 1 and 2. This ROS concentration threshold needed to trigger nanoceria activity is apparently found to be between M1 and M2 phenotypes. In such a way that during M1 polarization, ROS is efficiently scavenged by nanoceria, but no nanoceria activity or biological effect has been observed when cells are expressing an M0 or M2 phenotype. Indeed, it has been recurrently observed that the use of nanoceria enables the expression of M2 polarization and increased production of SOD, Arginase, or NOS synthetases (54), well known M2 enzymes and cytokines, employed to protect the cell from OS. This is probably not because nanoceria promotes M2 polarization. This is simply because when M1 polarization is stopped, M2 is allowed to take control of the produced damage and repair the tissue. In the opposite direction, nanoceria ROS buffering capacity increases with ROS concentration up to NP surface saturation. Taking into account that ROS have to arrive, absorb, react and desorb from the NP surface, surface saturation will determine the highest ROS concentration it can be managed at once. Consequently, the nanoceria scavenging reaction rate will be constant while ROS is in excess (according to the NP surface). This reaction rate will diminish as ROS concentration decreases until it stops, corresponding to homeostatic concentrations of ROS. This buffering capacity is at the origin of their immunomodulatory properties.

Nanoceria biodegradation can also be described with the above equations. Cerium oxide is known to be a non-biodegradable material. However, in its nanometric form, at neutral pH and low oxygen concentration, nanoceria in water thermodynamically prefers to stay in the Ce^3+^ soluble valence state rather than in the Ce^4+^ insoluble valence state (see Pourbaix diagram in SI (55)). Thus, during the catalytic cycle, an “activated” state, Ce^3+^, can leak from the NP and swim away. Consequently, *in vivo*, nanoceria can degrade into innocuous Ce^3+^ ions, expulsed from the body through the urine. The degradation of nanoceria during its biological action was reported for the first time in 2014, after observing how intracellular antioxidants dissolve man-made antioxidant nanoparticles and proposing to use the redox vulnerability of nanoceria to develop a responsive drug delivery system (56). Recent data show how nanoceria distributes, accumulates, and is expulsed from healthy mice after intravenous injection (54). At few hours after injection, the NPs are accumulated in the liver and spleen. From this point, the cerium concentration progressively decreases following an exponential decay where half of the dose has been expulsed in 6 weeks. During this experiment, cerium was found in urine and feces (57). Probably ions in the first case and NPs in the second, expulsed through the hepatobiliary route. This degradation of nanoceria can be promoted by increasing the reducing environment, decreasing pH (as in endolysosomes), and complexing molecules that absorb Ce^3+^ ions in solution and remove them from the equilibrium. As a final consideration, the smaller the NP, the higher their dissolution rate. Regarding nanoceria excretion, it is worthy to mention the observed excretion of nanoceria coated with dextran when administered orally as a computed tomography contrast agent for imaging the gastrointestinal tract in a mice model of Inflammatory Bowel Disease (58).

## Pharmacological Development of Nanoceria

These last two decades have started building a pharmacokinetic model for nanoceria, its behavior in physiological media, administration, biodistribution, degradation, and excretion. Still, today, the pharmacological knowledge on the subject is premature, mainly due to material uncertainty. First, NPs for medicine should be monodisperse, biocompatible, small, and highly dispersible in physiological media, with engineered surfaces to escape from phagocytosis (59). The traditional industrial basic precipitation employed for the preparation of nanoceria is normally continued by a calcination step to fully oxidize Ce^3+^ to Ce^4+^ and fully dehydrate Ce(OH)_4_ into CeO_2_. Nanoceria prepared in these conditions sinters and grows, losing its therapeutical properties, and needs high temperature to be activated, which is common in its industrial applications. Interestingly, current procedures for preparing nanoceria for medical applications by basic precipitation yield small colloidal Ce^+3/+4^ hydroxide/oxide NPs that can be employed in biology. The Ce^3+^/Ce^4+^ ratio is not always specified in the scientific literature despite being highly dependent on the NP size, preparation technique, NP history, and surface state. It can be determined by some characterization techniques, including X-ray photoelectron spectroscopy (XPS) (60), X-ray absorption spectroscopy (52), and UV-visible absorption spectroscopy (UV/VIS) (61).

We recommend the use of CeCl_3_ as cerium precursor instead of Ce(NO_3_)_3_ when using TMAOH as the base (chosen because of the stabilizing effect of TMA^+^ counterions) since the presence of nitrate in the synthesis process may derivate in nitrosamine contaminants which are of serious concerns for the regulatory agencies. In this respect, the “FDA guidance for industry on drug products, including biological products, that contain nanomaterials” (62) can be of great help in developing medical nanoceria. Related to that, a paper indicating how to carry pre-clinical studies on nanoceria harmonized with FDA regulations has recently been published (63). It is expected that nanoceria will have to follow the path other metal oxide inorganic NPs have followed, such as Fe_3_O_4_ NPs, approved as a contrast agent for MRI (Resovist^®^), as iron supply in the case of iron-deficiency anemia (Feromuxytol^®^) or as hyperthermia agent to treat neuroblastoma (Nanotherm^®^).

Special attention must be given to size, parental (as-synthesized), and eventually, aggregated (when dispersed). Aggregation, especially in physiological media, corresponds to the NP natural tendency to reduce surface area and consequently surface energy. Size is critical for both the catalytic activity of nanoceria and their pharmacological properties. This is because the number of oxygen vacancies increases with reducing the size and surface accesses increases for non-aggregate NPs, and because size is a major parameter of the administration, biodistribution, metabolization, and excretion profile of NPs (57).

The biodistribution of NPs is different from traditional small drugs designed to cross biological barriers and membranes, and distribute across the body. Nanoceria follows the main principles of NP biodistribution which depends on size, hydrophilicity, and surface charge (64). The initial observation is how reducing NP size extends blood circulating times and reaches good levels of homogeneous distribution in tissues (65). For neutral and negatively charged small inorganic NPs, depending on the portal of entry, different organs can be targeted. Normally, after i.v. administration, NPs accumulate in the liver (90%) and spleen (9%) after a few hours of blood circulation (66). However, NPs smaller than 6 nm can be rapidly cleared through the urinary tract (67). To avoid this, small nanoceria can be conjugated to different biomolecules, such as albumin (54), to prevent aggregation and avoid renal clearance. The reported most prolonged half-life in blood for nanoceria has been about 4 hours after injection (68). It is also feasible to target the lymph nodes after intramuscular injection, or the eye, the skin, and the gastrointestinal tract, by oral and topical administration. If injected into a tumor or the brain, NPs tend to remain inside the organ. It is also essential to consider that body barriers controlling NP dispersion are altered during the course of disease. Two significant cases are worthy of mention. First is the enhanced penetration and retention effect of NPs into solid tumors, described by Maeda *et al.* in 2002 (69), where due to abnormal angiogenesis, blood vessels supplying nutrients to solid tumors have defects in their tiling, and large pores (hundreds of nm) are formed, making the tumor accessible to nanocarriers, which together with poor lymphatic drainage, facilitate their accumulation (indeed albumin act as a nanometric carrier for cisplatin favoring its accumulation in tumors) (70). Second is the increase of barrier permeability during inflammation, as in the case of neuroinflammation, granting access to the brain to NPs after i.v. or i.p. injection (68, 71). All in all means that nanoceria can be designed to passively reach and stay in different organs for an extended period of time, depending on NP features, medical state, and administration route.

At the cellular level, NPs distribution has also been well described (72). For many different materials such as gold or iron oxide, NPs are found to be densely aggregated in endosomes persistent during the experimental times. The same is observed with nanoceria (54). In these cases, one would say that the nanoceria will not be functional because it is kept away from the cytoplasm, and this is true; however, as ROS can cross the phospholipid bilayers in the form of H_2_O_2_, the aggregate nanoceria can perform its task scavenging cytoplasmatic ROS that enters the endosome. Hypothetically, if long-lasting and functional, these structures could be pictorially called *ceriasomes*, a nanoceria highly loaded (hundreds of NPs) endosome which scavenges free radicals as soon as they enter, as artificial intracellular OS protective organelles. Besides, nanoceria permeation out of the endosome can increase during endosomal acidification if it loses its surface charge and become non-charged (depending on lysosome pH, nanoceria concentration, and nanoceria isoelectric point). In addition, proton-sponge-like effects due to its basic oxide surface may contribute to endosomal disruption. Nevertheless, this appears to happen only eventually. Therefore, most nanoceria will remain in endosomal vesicles inside the cytoplasm, acting as a ROS scavenging organelle.

These studies are, in part, possible thanks to the easy traceability of cerium. As a xenobiotic element, its background presence in the body is negligible, making it possible to trace its presence to attomolar concentrations by inductively coupled plasma mass spectroscopy (ICPMS). Additionally, thanks to its high Z number, it gives strong contrast, not only in optical and electron microscopies but also to X-ray (58). These aspects are not shared by conventional drugs that “disappear” as soon as they enter the body, and complex chemical and biochemical resolution-limited techniques must be employed.

Regarding dosing, nanoceria has been administered formulated with BSA (54), PEG (71), sodium citrate and EDTA (68), or a series of drugs (73). In most cases, aggregates of few tens nm have been employed, however, works with non aggregated NPs showed better biodistribution and increased biocompatibility. In any case, despite formulation and aggregation state, successful nanoceria applications work at concentrations of a few micrograms (50 to 250 µg) per gram of tissue, administered in single injections at concentrations of about 1 to 10 mg/ml, which in the case of 3 nm NPs corresponds to 9.25x10^15^ NPs/ml to 9.25x10^16^ NPs/ml. It is normally administered up to 1 mg nanoceria per Kg of animal, in 200 (mice) or 300 (rat) microliter volumes (**Table 2**, see an extended version in the SI, which includes formulation and administration route, among others).

**Table 2:** Dosing of nanoceria in different *in vivo* studies.

Study #	1 (74)	2 (75)	3 (76)	4 (77)	5 (78)	6 (79)	7 (68)	8 (80)	9 (81)	10 (82)	11 (71)
Size* (nm)	3	1, 3	10	No data	3	1-2.5	2.4	3	10	No data	3.3
Dose (µg/g)	No Data	No Data	0.05, 0.5, 5, 50	0.1	30, 100	10, 6	10, 20, 30	20	0.05, 0.5	0.05, 0.5, 5	0.1, 0.3, 0.5, 0.7, 1, 1.5

*parental size, independently of aggregation state.

### Nanosafety

Since the seminal work of Vicky Colvin in 2003 (56), nanosafety has been one of the most significant issues when discussing NP medical, industrial, and customer applications. Since then, a great effort has been dedicated to studying the detrimental aspects of NPs. Initial results were sometimes puzzling and confusing. In this regard, Prof. Harald Krug, in a 2014 review article (83), analyzing about 10.000 nanotoxicology papers, revealed that most of the nanotoxicity studies “do not offer a clear statement on the safety of nanomaterials and, on the contrary, most of them are either self-contradictory or arrive at completely erroneous conclusions.” Indeed, it has been observed that at realistic doses in a controlled manner, NPs show no significant increased toxicity compared to their molecular or bulk counterparts. The reported toxicity often has to be attributed to NPs aggregation and NPs association with toxic moieties (endotoxin, surfactants, or allergens) rather than the NPs themselves, leading to apparently contradictory data. Nanoceria is an illustrative case (84). While it is reported many times to be beneficial in protecting against oxidative stress and irradiation damage, other studies, mainly related to the toxicity of nanoceria in the industrial dry form (nanometric aggregate powders), show *in vitro* and *in vivo* toxicity (81). Similarly, while some studies show anti-inflammatory effects of nanoceria taken up by hepatocytes (54), others report liver macrophage (Kupffer cells) uptake and pro-inflammatory effects (85). This often results from the challenging dispersibility of inorganic NPs in physiological media that too often leads to NP aggregation and sedimentation, losing their beneficial properties. *In vitro* studies showed how the nanoceria ROS scavenging capacity increased with nanoceria concentration until it was lost when NPs aggregated at higher concentrations and started being pro-inflammatory (54). Interestingly, the therapeutic doses are far from these toxic doses (10 to 100 times). Large aggregates are easily detected by the immune system, and often a pro-inflammatory response is triggered, making the medical use of NPs complicated because of material uncertainty. Ji et al. demonstrated the importance of controlling NPs size, shape, and aggregation state. Inflammatory immune response and toxicity were only reported when using high aspect ratio nanoceria nanowires at high doses and aggregation state. Besides, it is important to note that some toxic ingredients coming from the NP formulation or derived from chemicals employed during NP preparation can misreport NP toxicity (83). An example is the work of Dowding et al. where a similar nanoceria synthesis process was done, but using different bases [NH_4_OH or hexamethylenetetramine (HMTA)] (86). Results showed that HMTA-nanoceria NPs were readily taken into endothelial cells and reduced cell viability at a 10-fold lower concentration than the other NPs, which showed no toxicity. Finally, a paper was published recently on a woman who drank a large amount of nanoceria-based polishing powder by mistake (87). The product was not described, but this industrial nanoceria is a mix of NP aggregates at a relatively high pH. The observed transient toxic effects could be related to the basic pH of the preparation and the presence of Ce^3+^ soluble species in the formulation. Besides, observed coagulation disorders had been previously described with Ce^3+^ at high doses because of their interference with Ca^2+^ homeostasis (88).

Therefore, well-described, pure, monodisperse, and highly dispersible in physiological media nanoceria is mandatory for this promising material meaningful and controlled use for therapy. One strategy to avoid nanoceria toxicity due to aggregation when dispersed in physiological media is pre-albuminization before injection (54). The albumin has not to be firmly bound to the NP. Its mere presence prevents NP aggregation by interacting with its surface. Once injected, NPs dilute in the bloodstream, or tissue, putting away aggregation risk.

Finally, cerium is affordable, abundant as silver (not in veins though, which makes its mining complicated), and nanoceria is easy to produce following green chemistry principles -at RT with simple reagents having recyclable basic waters as by-product. It is stable in simple storage conditions, and of universal use (the same NPs perform well in different disease models). Additionally, it is xenobiotic, which makes it easily traceable by imaging and spectroscopic techniques such as X-ray or mass spectroscopy, facilitating its preclinical studies. Thus, nanoceria and other nanozymes may represent a new era for medicine, where the ability to buffer excess ROS allows for better general population health (anti-aging, anti-tumoral and anti-inflammatory).

Summarizing, the relationship between metabolism and disease has been extensively explored during the past decade. Understanding how cells use energy to perform their functions has attracted attention concerning diseases such as obesity, diabetes, cancer, and neurodegeneration. Indeed, pathological inflammation is at the origin and progression of many diseases, from chronic, inducing accelerated aging and oncogenesis, to acute, such as ischemia, cytokine storms, and anaphylaxis. Antioxidant substances have shown promising immunomodulation in pre-clinical and epidemiological studies, and their mechanism has been observed in detail. However, they are still poorly translated to the clinic. The inconsistencies between the mechanistic and epidemiological studies, and the clinical trials, indicate the poor pharmacological properties of currently available substances and the need for new approaches and strategies. Today, nanoceria, catalytic mild antioxidant NPs, may provide the required pharmacokinetics and overcome previous limitations, unleashing the power of antioxidant prevention and therapy.

Cerium is a rare earth element that accumulates oxygen vacancies in its nanometric oxide form capable of catalytically removing excess ROS in metabolic imbalance situations. Indeed, nanoceria act as a redox buffer, promoting immunomodulation without immune suppression. Nanoceria displays a good safety profile to normal tissues while providing cellular protection from various forms of ROS and irradiation. Thanks to its catalytic nature, nanoceria can be used at low doses for a prolonged time (before NPs are degraded, dissolved, and excreted). Small nanoceria in the neutral pH and low oxidant conditions inside the body slowly dissolves in few months as the insoluble Ce^4+^ is progressively reduced to soluble Ce^3+^ ions, excreted through the urine. Nanoceria is redox selective (only degrades ROS at high concentrations) but not ROS selective (degrades any form of ROS). Indeed, it is selective to a high concentration of unpaired electrons regardless of the atomic orbital carrying them. When adequately formulated (endotoxin-free, stable, soluble), no harmful effects have been observed in *in vitro* and *in vivo* models at applicable doses. Of note, cerium compounds (cerium oxalate and cerium nitrate) were used in the past, among others, as antiemetic agents during pregnancy. Nanoceria formulation and dose will have to be developed case by case since tissue environment, and the metabolic and immune status depends on the studied tissue and medical condition. For example, the brain, despite its high consumption of glucose and tendency to suffer from oxidative stress, it is short in endogenous antioxidant defenses. Or like pregnancy, which starts with the immune system activating an M1 polarization to follow up with an M2 polarization from placentation up to delivery.

## Data Availability Statement

The original contributions presented in the study are included in the article/**Supplementary Material**. Further inquiries can be directed to the corresponding author.

## Author Contributions

LM and VP developed the hypothesis, search the literature, wrote the manuscript and approved it for publication.

## Conflict of Interest

The authors declare that the research was conducted in the absence of any commercial or financial relationships that could be construed as a potential conflict of interest.

## Publisher’s Note

All claims expressed in this article are solely those of the authors and do not necessarily represent those of their affiliated organizations, or those of the publisher, the editors and the reviewers. Any product that may be evaluated in this article, or claim that may be made by its manufacturer, is not guaranteed or endorsed by the publisher.

## References

[1] Liemburg-Apers DC Willems PHGM Koopman WJH Grefte S. Interactions Between Mitochondrial Reactive Oxygen Species and Cellular Glucose Metabolism. Arch Toxicol (2015) 89:1209–26. doi: 10.1007/s00204-015-1520-y PMC4508370 26047665

[2] Viola A Munari F Sánchez-Rodríguez R Scolaro T Castegna A. The Metabolic Signature of Macrophage Responses. Front Immunol (2019) 10:1462. doi: 10.3389/fimmu.2019.01462 31333642 PMC6618143

[3] Arulselvan P Fard MT Tan WS Gothai S Fakurazi S Norhaizan ME. Role of Antioxidants and Natural Products in Inflammation. Oxid Med Cell Longev (2016) 2016:5276130. doi: 10.1155/2016/5276130 27803762 PMC5075620

[4] Liguori I Russo G Curcio F Bulli G Aran L Della-Morte D. Oxidative Stress, Aging, and Diseases. Clin Interv Aging (2018) 13:757–72. doi: 10.2147/CIA.S158513 PMC5927356 29731617

[5] Pauling L. Vitamin C and the Common Cold. Can Med Assoc J (1971) 105:448. doi: 10.1001/jama.1971.03180280086025

[6] Vitamin C and the Common Cold (1970). Available at: https://www.worldcat.org/title/vitamin-c-and-the-common-cold/oclc/107441 (Accessed June 6, 2021). WorldCat.org.

[7] Knekt P Reunanen A Jävinen R Seppänen R Heliövaara M Aromaa A. Antioxidant Vitamin Intake and Coronary Mortality in a Longitudinal Population Study. Am J Epidemiol (1994) 139:1180–9. doi: 10.1093/oxfordjournals.aje.a116964 8209876

[8] Natural Antioxidants in Human Health and Disease - Google Llibres . Available at: https://books.google.es/books?hl=ca&lr=&id=GYUXAAAAQBAJ&oi=fnd&pg=PP1&dq=antioxidant+cancer+inflammation+neuro*&ots=vR9knTlVcL&sig=j30lop6HKgW1D_FwKzmVbyceSiw#v=onepage&q&f=false (Accessed June 30, 2021).

[9] Steinhubl SR. Why Have Antioxidants Failed in Clinical Trials? Am J Cardiol (2008) 101:S14–9. doi: 10.1016/j.amjcard.2008.02.003 18474268

[10] Firuzi O Miri R Tavakkoli M Saso L. Antioxidant Therapy: Current Status and Future Prospects. Curr Med Chem (2012) 18:3871–88. doi: 10.2174/092986711803414368 21824100

[11] Benfeito S Oliveira C Soares P Fernandes C Silva T Teixeira J. Antioxidant Therapy: Still in Search of the “Magic Bullet.” Mitochondrion (2013) 13:427–35. doi: 10.1016/j.mito.2012.12.002 23246773

[12] Xu C Qu X. Cerium Oxide Nanoparticle: A Remarkably Versatile Rare Earth Nanomaterial for Biological Applications. NPG Asia Mater (2014) 6:e90–0. doi: 10.1038/am.2013.88

[13] Casals E Zeng M Parra-Robert M Fernández-Varo G Morales-Ruiz M Jiménez W. Cerium Oxide Nanoparticles: Advances in Biodistribution, Toxicity, and Preclinical Exploration. Small (2020) 16:1907322. doi: 10.1002/smll.201907322 32329572

[14] Willner D Weissman C. Carbon Dioxide Production, Metabolism, and Anesthesia. In: Capnography, 2nd ed. Cambridge: Cambridge University Press. (2011). p. 239–49. doi: 10.1017/CBO9780511933837.026

[15] Klingenberg M Rottenberg H. Relation Between the Gradient of the ATP/ADP Ratio and the Membrane Potential Across the Mitochondrial Membrane. Eur J Biochem (1977) 73:125–30. doi: 10.1111/j.1432-1033.1977.tb11298.x 14003

[16] Liberti MV Locasale JW. The Warburg Effect: How Does it Benefit Cancer Cells? Trends Biochem Sci (2016) 41:211. doi: 10.1016/J.TIBS.2015.12.001 26778478 PMC4783224

[17] Magnasco MO. Molecular Combustion Motors. Phys Rev Lett (1994) 72:2656. doi: 10.1103/PhysRevLett.72.2656 10055939

[18] Shen Y Kapfhamer D Minnella AM Kim JE Won SJ Chen Y. Bioenergetic State Regulates Innate Inflammatory Responses Through the Transcriptional Co-Repressor CtBP. Nat Commun (2017) 8:1–13. doi: 10.1038/s41467-017-00707-0 28935892 PMC5608947

[19] Karbowski M Youle RJ. Dynamics of Mitochondrial Morphology in Healthy Cells and During Apoptosis. Cell Death Differ (2003) 10:870–80. doi: 10.1038/sj.cdd.4401260 12867994

[20] Zhou J Deo BK Hosoya K Terasaki T Obrosova IG Brosius FC. Increased JNK Phosphorylation and Oxidative Stress in Response to Increased Glucose Flux Through Increased GLUT1 Expression in Rat Retinal Endothelial Cells. Investig Ophthalmol Vis Sci (2005) 46:3403–10. doi: 10.1167/iovs.04-1064 16123445

[21] Talior I Yarkoni M Bashan N Eldar-Finkelman H. Increased Glucose Uptake Promotes Oxidative Stress and PKC-δ Activation in Adipocytes of Obese, Insulin-Resistant Mice. Am J Physiol - Endocrinol Metab (2003) 285:295–02. doi: 10.1152/ajpendo.00044.2003 12857675

[22] Brand KA Hermfisse U. Aerobic Glycolysis by Proliferating Cells: A Protective Strategy Against Reactive Oxygen Species 1. FASEB J (1997) 11:388–95. doi: 10.1096/fasebj.11.5.9141507 9141507

[23] Yang S Lian G. ROS and Diseases: Role in Metabolism and Energy Supply. Mol Cell Biochem (2020) 467:1. doi: 10.1007/s11010-019-03667-9 31813106 PMC7089381

[24] Di Meo S Reed TT Venditti P Victor VM. Role of ROS and RNS Sources in Physiological and Pathological Conditions. Oxid Med Cell Longev (2016) 2016:1245049. doi: 10.1155/2016/1245049 27478531 PMC4960346

[25] Van Anders G Klotsa D Ahmed NK Engel M Glotzer SC. Understanding Shape Entropy Through Local Dense Packing. Proc Natl Acad Sci U.S.A. (2014) 111:E4812–21. doi: 10.1073/pnas.1418159111 PMC4234574 25344532

[26] deS Breda CN Davanzo GG Basso PJ Saraiva Câmara NO Moraes-Vieira PMM. Mitochondria as Central Hub of the Immune System. Redox Biol (2019) 26:101255. doi: 10.1016/j.redox.2019.101255 31247505 PMC6598836

[27] Chaplin DD. Overview of the Immune Response. J Allergy Clin Immunol (2010) 125:S3–S23. doi: 10.1016/j.jaci.2009.12.980 20176265 PMC2923430

[28] Italiani P Boraschi D. From Monocytes to M1/M2 Macrophages: Phenotypical vs. Functional Differentiation. Front Immunol (2014) 5:514. doi: 10.3389/fimmu.2014.00514 25368618 PMC4201108

[29] Andersson SGE Karlberg O Canbäck B Kurland CG Whatley FR van der Giezen M. On the Origin of Mitochondria: A Genomics Perspective. Philos Trans R Soc B: Biol Sci (Royal Society) (2003) 358:165–79. doi: 10.1098/rstb.2002.1193 PMC1693097 12594925

[30] O’Neill LAJ Kishton RJ Rathmell J. A Guide to Immunometabolism for Immunologists. Nat Rev Immunol (2016) 16:553–65. doi: 10.1038/nri.2016.70 PMC5001910 27396447

[31] The Medical Times and Gazette. New Ser.:V.19 (1859). HathiTrust Digital Library | HathiTrust Digital Library. Available at: https://babel.hathitrust.org/cgi/pt?id=hvd.32044103088555&view=1up&seq=288 (Accessed June 1, 2021).

[32] Simpson JY. Note on the Therapeutic Action of the Salts of Cerium. Mon J Med Sci (1854) 10:563.

[33] Baehr G Wessler H. The Use of Cerium Oxalate for the Relief of Vomiting: An Experimental Study of the Effects of Some Salts of Cerium, Lanthanum, Praseodymium, Neodymium and Thorium. Arch Intern Med (1909) II:517–31. doi: 10.1001/archinte.1909.00050110014002

[34] Rice KM Bandarupalli VVK Manne NDPK Blough ER. Spleen Data: Cerium Oxide Nanoparticles Attenuate Polymicrobial Sepsis Induced Spenic Damage in Male Sprague Dawley Rats. Data Br (2018) 18:740–6. doi: 10.1016/j.dib.2018.03.073 PMC5996310 29900230

[35] Van Noorden CJF Frederiks WM. Cerium Methods for Light and Electron Microscopical Histochemistry. J Microsc (1993) 171:3–16. doi: 10.1111/j.1365-2818.1993.tb03354.x 8396182

[36] Veenhuis M Bonga SEW. Cytochemical Localization of Catalase and Several Hydrogen Peroxide-Producing Oxidases in the Nucleoids and Matrix of Rat Liver Peroxisomes. Histochem J (1979) 11:561–72. doi: 10.1007/BF01012539 511592

[37] Vaughn KC Duke SO Duke SH Henson CA. Ultrastructural Localization of Urate Oxidase in Nodules of Sesbania Exaltata, Glycine Max, and Medicago Sativa. Histochemistry (1982) 74:309–18. doi: 10.1007/BF00493430 7201988

[38] Veenhuis M Van Dijken JP Harder W. A New Method for the Cytochemical Demonstration of Phosphatase Activities in Yeasts Based on the Use of Cerous Ions. FEMS Microbiol Lett (1980) 9:285–91. doi: 10.1111/j.1574-6968.1980.tb05654.x

[39] Telek G Scoazec JY Chariot J Ducroc R Feldmann G Rozé C. Cerium-Based Histochemical Demonstration of Oxidative Stress in Taurocholate-Induced Acute Pancreatitis in Rats: A Confocal Laser Scanning Microscopic Study. J Histochem Cytochem (1999) 47:1201–12. doi: 10.1177/002215549904700912 10449541

[40] Rzigalinski BA Bailey D Chow L Kuiry SC Patil S Merchant S. Cerium Oxide Nanoparticles Increase the Lifespan of Cultured Brain Cells and Protect Against Free Radical and Mechanical Trauma. FASEB J (2003) 17.

[41] Rzigalinski BA. Nanoparticles and Cell Longevity. Techonology Cancer Res Treat (2005) 4:651–9. doi: 10.1177/153303460500400609 16292885

[42] Cox GM Harrison TS McDade HC Taborda CP Heinrich G Casadevall A. Superoxide Dismutase Influences the Virulence of Cryptococcus Neoformans by Affecting Growth Within Macrophages. Infect Immun (2003) 71:173–80. doi: 10.1128/IAI.71.1.173-180.2003 PMC143417 12496163

[43] Hashem RM Rashd LA Hashem KS Soliman HM. Cerium Oxide Nanoparticles Alleviate Oxidative Stress and Decreases Nrf-2/HO-1 in D-GALN/LPS Induced Hepatotoxicity. BioMed Pharmacother (2015) 73:80–6. doi: 10.1016/j.biopha.2015.05.006 26211586

[44] Allawadhi P Khurana A Allwadhi S Joshi K Packirisamy G Bharani KK. Nanoceria as a Possible Agent for the Management of COVID-19. Nano Today (2020) 35:100982. doi: 10.1016/j.nantod.2020.100982 32952596 PMC7492057

[45] Finkel T Holbrook NJ. Oxidants, Oxidative Stress and the Biology of Ageing. Nature (2000) 408:239–47. doi: 10.1038/35041687 11089981

[46] Chen J Patil S Seal S McGinnis JF. Rare Earth Nanoparticles Prevent Retinal Degeneration Induced by Intracellular Peroxides. Nat Nanotechnol (2006) 1:142–50. doi: 10.1038/nnano.2006.91 18654167

[47] D’Angelo B Santucci S Benedetti E Di Loreto S Phani R Falone S. Cerium Oxide Nanoparticles Trigger Neuronal Survival in a Human Alzheimer Disease Model By Modulating BDNF Pathway. Curr Nanosci (2009) 5:167–76. doi: 10.2174/157341309788185523

[48] Pinna A Malfatti L Galleri G Manetti R Cossu S Rocchitta G. Ceria Nanoparticles for the Treatment of Parkinson-Like Diseases Induced by Chronic Manganese Intoxication. RSC Adv (2015) 5:20432–9. doi: 10.1039/C4RA16265J

[49] Carvajal S Perramón M Oró D Casals E Fernández-Varo G Casals G. Cerium Oxide Nanoparticles Display Antilipogenic Effect in Rats With non-Alcoholic Fatty Liver Disease. Sci Rep (2019) 9:1–20. doi: 10.1038/s41598-019-49262-2. 2019 91. 31492960 PMC6731222

[50] Córdoba-Jover B Arce-Cerezo A Ribera J Pauta M Oró D Casals G. Cerium Oxide Nanoparticles Improve Liver Regeneration After Acetaminophen-Induced Liver Injury and Partial Hepatectomy in Rats. J Nanobiotechnol (2019) 17:1–12. doi: 10.1186/S12951-019-0544-5. 2019 171. PMC6822381 31672158

[51] Reed K Cormack A Kulkarni A Mayton M Sayle D Klaessig F. Exploring the Properties and Applications of Nanoceria: Is There Still Plenty of Room at the Bottom? Environ Sci Nano (2014) 1:390–405. doi: 10.1039/C4EN00079J

[52] Cafun J-D Kvashnina KO Casals E Puntes VF Glatzel P. Absence of Ce3+ Sites in Chemically Active Colloidal Ceria Nanoparticles. ACS Nano (2013) 7:10726–32. doi: 10.1021/nn403542p 24215500

[53] Das S Singh S Dowding JM Oommen S Kumar A Sayle TXT. The Induction of Angiogenesis by Cerium Oxide Nanoparticles Through the Modulation of Oxygen in Intracellular Environments. Biomaterials (2012) 33:7746–55. doi: 10.1016/J.BIOMATERIALS.2012.07.019 PMC4590782 22858004

[54] Oró D Yudina T Fernández-Varo G Casals E Reichenbach V Casals G. Cerium Oxide Nanoparticles Reduce Steatosis, Portal Hypertension and Display Anti-Inflammatory Properties in Rats With Liver Fibrosis. J Hepatol (2016) 64:691–8. doi: 10.1016/j.jhep.2015.10.020 26519601

[55] Channei D Phanichphant S Nakaruk A Mofarah SS Koshy P Sorrell CC. Aqueous and Surface Chemistries of Photocatalytic Fe-Doped CeO2 Nanoparticles. Catal (2017) 7:45. doi: 10.3390/CATAL7020045

[56] Muhammad F Wang A Qi W Zhang S Zhu G. Intracellular Antioxidants Dissolve Man-Made Antioxidant Nanoparticles: Using Redox Vulnerability of Nanoceria to Develop a Responsive Drug Delivery System. ACS Appl Mater Interfaces (2014) 6:19424–33. doi: 10.1021/am5055367 25312332

[57] Poon W Zhang YN Ouyang B Kingston BR Wu JLY Wilhelm S. Elimination Pathways of Nanoparticles. ACS Nano (2019) 13:5785–98. doi: 10.1021/ACSNANO.9B01383 30990673

[58] Naha PC Hsu JC Kim J Shah S Bouché M Si-Mohamed S. Dextran-Coated Cerium Oxide Nanoparticles: A Computed Tomography Contrast Agent for Imaging the Gastrointestinal Tract and Inflammatory Bowel Disease. ACS Nano (2020) 14:10187–97. doi: 10.1021/ACSNANO.0C03457 PMC7484129 32692538

[59] Ernst LM Casals E Italiani P Boraschi D Puntes V. The Interactions Between Nanoparticles and the Innate Immune System From a Nanotechnologist Perspective. Nanomater (2021) 11:2991. doi: 10.3390/NANO11112991. 2021, Vol 11, Page 2991. PMC8621168 34835755

[60] Garcia X Soler L Divins NJ Vendrell X Serrano I Lucentini I. Ceria-Based Catalysts Studied by Near Ambient Pressure X-Ray Photoelectron Spectroscopy: A Review. Catal (2020) 10:286. doi: 10.3390/CATAL10030286. 2020, Vol 10, Page 286.

[61] Manoharan D. Optical Properties of Nano-Crystalline Cerium Dioxide Synthesized by Single Step Aqueous Citrate-Nitrate Gel Combustion Method. Artic Asian J Chem (2013) 25:9045–49. doi: 10.14233/ajchem.2013.14984

[62] Fda Cder Yeaton Ayse. Drug Products, Including Biological Products, That Contain Nanomaterials - Guidance for Industry. Available at: https://www.fda.gov/Drugs/GuidanceComplianceRegulatoryInformation/Guidances/default.htm (Accessed July 6, 2021).

[63] Ghorbani M Izadi Z Jafari S Casals E Rezaei F Aliabadi A. Preclinical Studies Conducted on Nanozyme Antioxidants: Shortcomings and Challenges Based on US FDA Regulations. Futur Med (2021) 16:1133–51. doi: 10.2217/NNM-2021-0030 33973797

[64] McNeil SE. Nanoparticle Therapeutics: A Personal Perspective. Wiley Interdiscip Rev Nanomed Nanobiotechnol (2009) 1:264–71. doi: 10.1002/WNAN.6 20049796

[65] Hoshyar N Gray S Han H Bao G. The Effect of Nanoparticle Size on *In Vivo* Pharmacokinetics and Cellular Interaction. Nanomedicine (2016) 11:673. doi: 10.2217/NNM.16.5 27003448 PMC5561790

[66] Tsoi KM Macparland SA Ma XZ Spetzler VN Echeverri J Ouyang B. Mechanism of Hard-Nanomaterial Clearance by the Liver. Nat Mater (2016) 15:1212–21. doi: 10.1038/nmat4718 PMC5132626 27525571

[67] Soo Choi H Liu W Misra P Tanaka E Zimmer JP Itty Ipe B. Renal Clearance of Quantum Dots. Nat Biotechnol (2007) 25:1165–70. doi: 10.1038/nbt1340 PMC2702539 17891134

[68] Heckman KL Decoteau W Estevez A Reed KJ Costanzo W Sanford D. Custom Cerium Oxide Nanoparticles Protect Against a Free Radical Mediated Autoimmune Degenerative Disease in the Brain. ACS Nano (2013) 7:10582–96. doi: 10.1021/nn403743b 24266731

[69] Maeda H Wu J Sawa T Matsumura Y Hori K. Tumor Vascular Permeability and the EPR Effect in Macromolecular Therapeutics: A Review. J Control Release (2000) 65:271–84. doi: 10.1016/S0168-3659(99)00248-5 10699287

[70] Comenge J Sotelo C Romero F Gallego O Barnadas A Parada TGC. Detoxifying Antitumoral Drugs *via* Nanoconjugation: The Case of Gold Nanoparticles and Cisplatin. PloS One (2012) 7:47562. doi: 10.1371/journal.pone.0047562 PMC3474726 23082177

[71] Kim CK Kim T Choi I-Y Soh M Kim D Kim Y-J. Ceria Nanoparticles That can Protect Against Ischemic Stroke. Angew Chemie Int Ed (2012) 51:11039–43. doi: 10.1002/anie.201203780 22968916

[72] Sousa De Almeida M Susnik E Drasler B Taladriz-Blanco P Petri-Fink A Rothen-Rutishauser B. Understanding Nanoparticle Endocytosis to Improve Targeting Strategies in Nanomedicine. Chem Soc Rev (2021) 50:5397–434. doi: 10.1039/D0CS01127D PMC8111542 33666625

[73] Bao Q Hu P Xu Y Cheng T Wei C Pan L. Simultaneous Blood–Brain Barrier Crossing and Protection for Stroke Treatment Based on Edaravone-Loaded Ceria Nanoparticles. ACS Nano (2018) 12:6794–805. doi: 10.1021/ACSNANO.8B01994 29932327

[74] Kwon HJ Cha MY Kim D Kim DK Soh M Shin K. Mitochondria-Targeting Ceria Nanoparticles as Antioxidants for Alzheimer’s Disease. ACS Nano (2016) 10:2860–70. doi: 10.1021/ACSNANO.5B08045 26844592

[75] Wahba SMR Darwish AS Kamal SM. Ceria-Containing Uncoated and Coated Hydroxyapatite-Based Galantamine Nanocomposites for Formidable Treatment of Alzheimer’s Disease in Ovariectomized Albino-Rat Model. Mater Sci Eng C (2016) 65:151–63. doi: 10.1016/j.msec.2016.04.041 27157738

[76] Dillon CD Billings M Hockey KS Delagarza L Rzigalinski BA. Cerium Oxide Nanoparticles Protect Against MPTP-Induced Dopaminergic Neurodegeneration In A Mouse Model For Parkinson’s Disease. NSTI-Nanotech (2011) 3:451–4.

[77] Hegazy MAE Maklad HM Abd Elmonsif DA Elnozhy FY Alqubiea MA Alenezi FA. The Possible Role of Cerium Oxide (CeO2) Nanoparticles in Prevention of Neurobehavioral and Neurochemical Changes in 6-Hydroxydopamine-Induced Parkinsonian Disease. Alexandria J Med (2017) 53:351–60. doi: 10.1016/J.AJME.2016.12.006

[78] Kwon HJ Kim D Seo K Kim YG Han SI Kang T. Ceria Nanoparticle Systems for Selective Scavenging of Mitochondrial, Intracellular, and Extracellular Reactive Oxygen Species in Parkinson’s Disease. Angew Chemie Int Ed (2018) 57:9408–12. doi: 10.1002/anie.201805052 29862623

[79] Ceria Nanopartciles Reduce Disease Severity in a Mouse Model of Multiple Sclerosis – TechConnect Briefs . Available at: https://briefs.techconnect.org/papers/ceria-nanopartciles-reduce-disease-severity-in-a-mouse-model-of-multiple-sclerosis/ (Accessed July 21, 2021).

[80] DeCoteau W Heckman KL Estevez AY Reed KJ Costanzo W Sandford D. Cerium Oxide Nanoparticles With Antioxidant Properties Ameliorate Strength and Prolong Life in Mouse Model of Amyotrophic Lateral Sclerosis. Nanomed Nanotechnol Biol Med (2016) 12:2311–20. doi: 10.1016/J.NANO.2016.06.009 27389143

[81] Bailey ZS Nilson E Bates JA Oyalowo A Hockey KS Sajja VSSS. Cerium Oxide Nanoparticles Improve Outcome After *In Vitro* and *In Vivo* Mild Traumatic Brain Injury. J Neurotrauma (2016) 37:142–62. doi: 10.1089/neu.2016.4644. neu.2016.4644. PMC7249477 27733104

[82] Hicks HJ Jackson P Willner J Whiting M. Post-Injury Administration of Cerium Oxide Nanoparticles: A Dose-Response Study. (2013).

[83] Krug HF. Nanosafety Research-Are We on the Right Track? Angew Chemie Int Ed (2014) 53:12304–19. doi: 10.1002/anie.201403367 25302857

[84] Casals E Gusta MF Piella J Casals G Jiménez W Puntes V. Intrinsic and Extrinsic Properties Affecting Innate Immune Responses to Nanoparticles: The Case of Cerium Oxide. Front Immunol (2017) 8:970. doi: 10.3389/fimmu.2017.00970 28855907 PMC5557789

[85] Hirst SM Karakoti A Singh S Self W Tyler R Seal S. Bio-Distribution and *In Vivo* Antioxidant Effects of Cerium Oxide Nanoparticles in Mice. Environ Toxicol (2013) 28:107–18. doi: 10.1002/tox.20704 21618676

[86] Dowding JM Das S Kumar A Dosani T McCormack R Gupta A. Cellular Interaction and Toxicity Depend on Physicochemical Properties and Surface Modification of Redox-Active Nanomaterials. ACS Nano (2013) 7:4855–68. doi: 10.1021/NN305872D/SUPPL_FILE/NN305872D_SI_001.PDF PMC3700371 23668322

[87] Lu YQ. Coagulation Disorders Following an Accidental Ingestion of Cerium Dioxide Nanoparticles. Environ Toxicol Pharmacol (2021) 82:103560. doi: 10.1016/J.ETAP.2020.103560 33290874

[88] Environmental Protection Agency. Toxicological Review of Cerium Oxide and Cerium Compounds: In Support of Summary Information on the Integrated Risk Information System (IRIS). Fed Regist (2009).

