# Supplementary material for: How Does Immunomodulatory Nanoceria Work? ROS and Immunometabolism
Source: Front Immunol. 2022 Mar 17;13:750175. doi: 10.3389/fimmu.2022.750175 (PMC8989015; doi:10.3389/fimmu.2022.750175)
Supplement: Supplementary file 1 [file DataSheet_1.pdf]

How does immunomodulatory nanoceria works? ROS and immunometabolism.

Lena M. Ernst[1] *and Victor Puntes*[1,2,3,4]

[1] *Vall d'Hebron Research Instiute (VHIR), 08035 Barcelona, Spain*

[2] *Instiut Català de Nanociència I Nanotecnologia (ICN2), CSIC, The Barcelona Institute of Science and Technology (BIST), Campus UAB, Bellaterra, 08193 Barcelona, Spain*

[3] *Institució Catalana de Recerca I Estudis Avançats (ICREA), 08010 Barcelona, Spain*

[4] *Networking Research Centre for Bioengineering, Biomaterials, and Nanomedicine (CIBER-BBN), Instituto de Salud Carlos III, Madrid, Spain*

SUPPLEMENTARY INFORMATION

SUPPLEMENTARY TABLE OF NANOCERIA DOSING.

Study #	Nanoceria size (nm)	Dose (µg/g)	Coating/Stabilization	Hydrodynamic diameter (nm)	Z-Pot (mV)	Administration route	Therapeutic/Preventive regimen
**1** [75]	3	No data	DSPE-PEG	11	-23	Hippocampal injection	Single injection
			DSPE-PEG-TPP	22	45		
			FITC-DSPE-PEG-TPP	22	44		
**2** [76]	Crystallized nanoceria containing carboxymethyl chitosan 1 nm	No data	Tretragonal shaped HAp (70 nm length & 10 nm diameter) coated with CNPs, Galantamine and Carboxymethyl chitosan	480	3	Intramuscular injection	Daily administration for 1 month (two months after disease induction)
	Crystallized nanoceria 3 nm	No data	HAp rod-like NPs (85nm length & 5 nm diameter) coated with CNPs and Galantamine	112 & 650	-15-20	Intramuscular injection	Daily administration for 1 month (two months after disease induction)
**3** [77]	10	0.05<br>0.5<br>5<br>50	None	No data	No data	Intravenous Injection	X1 injection 5 days prior to disease induction, x3 injections along 3 days
**4** [78]	No data	0.1	No data	No data	No data	Intraperitoneal injection	1 injection 2 h before surgery and once daily for 6 weeks
**5** [79]	3	30	PEG	11	-23	Stereotaxic injection	1 single injection 1 day after disease induction
		30	TPP-conjugated PEG	22	45		
		100	Assemble 100000 CNPs	400	-20		

**6** [80]	1-2.5	10 µg /g before induction	None	No data	No data	Intravenous Injection	x1 injection 1 day before disease induction, x1 injection the day of induction, and x4 injections day 3, 7, 14 and 21 post induction
		6 µg /g after induction					x4 injections at day 3, 7, 14 and 21 post induction
**7** [68]	2.4	10<br>20<br>30	Citrate-EDTA stabilized	2.9	-23.5	Intravenous Injection	x1 injection 1 day before disease induction, x1 injection the day of induction, and x5 injections at day 7, 14, 21, 28 and 35 post induction
		10<br>20					x1 injection day 3 after induction and x5 injections at day 7, 14, 21, 28 and 35 post induction
		30					x1 injection day 7 after induction and x4 injections at day 14, 21, 28 and 35 post induction
**8** [81]	3	20	Citrate-EDTA stabilized	3.3	-22.94	Intravenous Injection	x2 injections weekly after development of muscle weakness until death
**9** [82]	10	0.05<br>0.5	none	No data	No data	Intravenous Injection	x3 injections over 3h post-injury
							x5 injections over 48h post-injury
**10** [83]	No data	0.05<br>0.5<br>5	No data	No data	No data	Intravenous Injection	Single injection 30 min after injury
**11** [71]	3.3	0.1<br>0.3<br>0.5<br>0.7<br>1<br>1.5	PEG	18-30	No data	Intravenous Injection	Single injection after injury

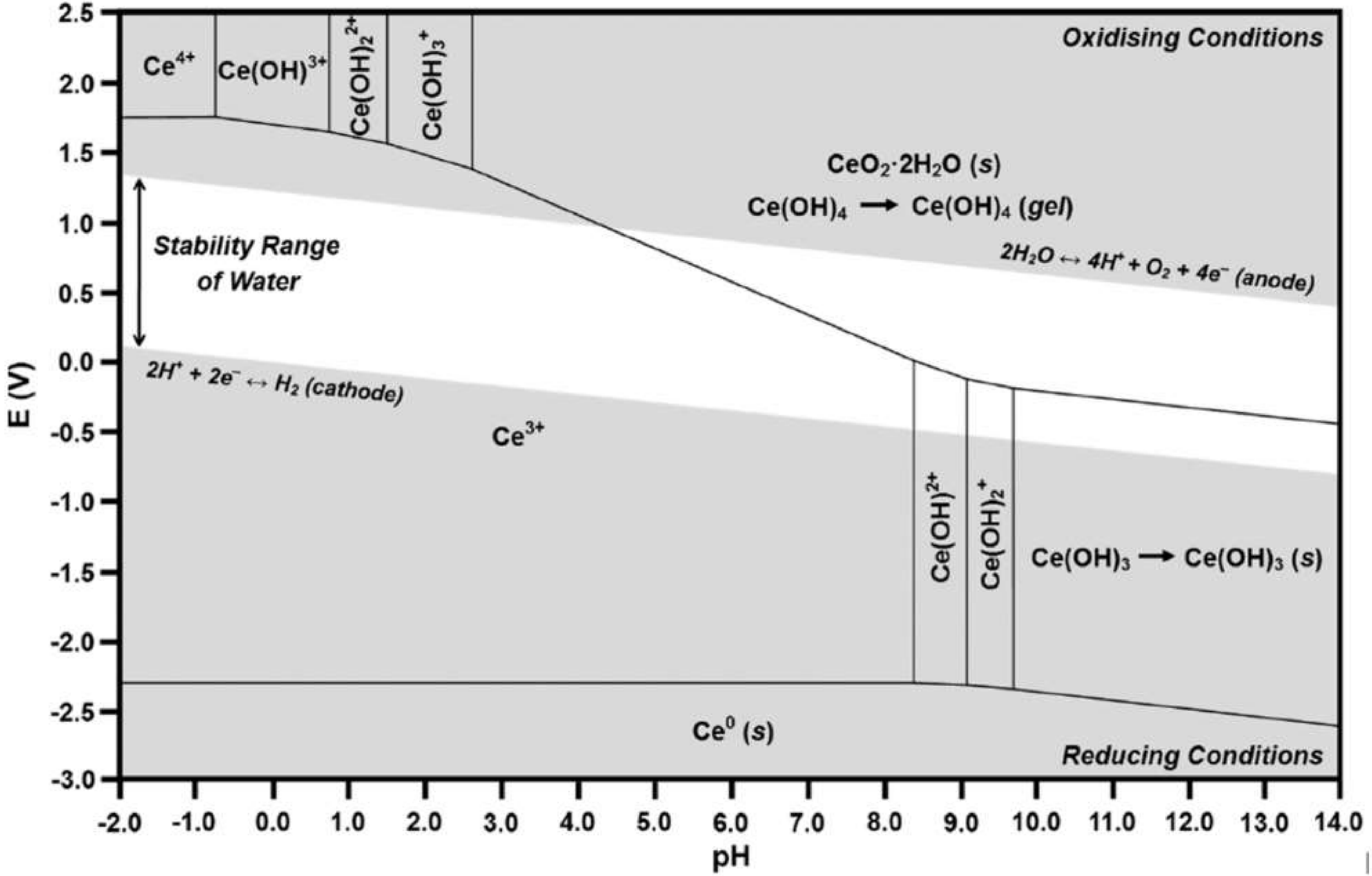

Oxidising Conditions
Ce^{4+}
$Ce(OH)^{3+}$
$Ce(OH)_2^{2+}$
$Ce(OH)_3^{+}$
$CeO_2 \cdot 2H_2O$ (s)
$Ce(OH)_4$ → $Ce(OH)_4$ (gel)
$2H_2O \leftrightarrow 4H^+ + O_2 + 4e^-$ (anode)
Stability Range of Water
$2H^+ + 2e^- \leftrightarrow H_2$ (cathode)
Ce^{3+}
$Ce(OH)^{2+}$
$Ce(OH)_2^{+}$
$Ce(OH)_3$ → $Ce(OH)_3$ (s)
Ce^0 (s)
Reducing Conditions
E (V)
pH
2.5
2.0
1.5
1.0
0.5
0.0
-0.5
-1.0
-1.5
-2.0
-2.5
-3.0
-2.0
-1.0
0.0
1.0
2.0
3.0
4.0
5.0
6.0
7.0
8.0
9.0
10.0
11.0
12.0
13.0
14.0
